# Stapleless laparoscopic left lateral sectionectomy for hepatocellular carcinoma: reappraisal of the Louisville statement by a young liver surgeon

**DOI:** 10.1186/s12876-018-0903-y

**Published:** 2018-11-28

**Authors:** Chao-Wei Lee, Hsin-I Tsai, Hao-Tsai Cheng, Wei-Ting Chen, Heng-Yuan Hsu, Chien-Chih Chiu, Yi-Ping Liu, Tsung-Han Wu, Ming-Chin Yu, Wei-Chen Lee, Miin-Fu Chen

**Affiliations:** 1 Department of Surgery, Linkou Chang Gung Memorial Hospital, No.5, Fuxing St, Guishan Dist, Taoyuan City, 33305 Taiwan, Republic of China; 2 College of Medicine, Chang Gung University, Guishan, Taoyuan Taiwan, Republic of China; 3 Graduate Institute of Clinical Medical Sciences, Chang Gung University, Guishan, Taoyuan Taiwan, Republic of China; 4 Department of Anesthesiology, Linkou Chang Gung Memorial Hospital, No.5, Fuxing St, Guishan Dist, Taoyuan City, 33305 Taiwan, Republic of China; 5 Department of Gastroenterology and Hepatology, Linkou Chang Gung Memorial Hospital, No.5, Fuxing St, Guishan Dist, Taoyuan City, 33305 Taiwan, Republic of China; 6 Department of Nursing, Linkou Chang Gung Memorial Hospital, No.5, Fuxing St, Guishan Dist, Taoyuan City, 33305 Taiwan, Republic of China; 7 Department of Surgery, Xiamen Chang Gung Hospital, Xiamen, China

**Keywords:** Laparoscopic hepatectomy, Liver resection, Left lateral sectionectomy, Hepatocellular carcinoma, Hepatoma, Stapleless, Louisville statement, Laparoscopic surgery, Hepatectomy, Young surgeon

## Abstract

**Background:**

Laparoscopic liver resection has been regarded as the standard treatment for liver tumors located at the left lateral liver sector. However, few studies have reported the results of laparoscopic left lateral sectionectomy (LLS) for HCC, not to mention the feasibility of this emerging technique for the less experienced liver surgeons. The current study would reappraise the Louisville statement by examining the outcome of LLS performed by a young liver surgeon.

**Methods:**

We retrospectively reviewed two separate groups of patients who underwent open or laparoscopic left lateral sectionectomies at Chung Gung Memorial Hospital, Linkou. All laparoscopic hepatectomies were performed by the index young surgeon following a stepwise stapleless LLS. The surgical results and oncological outcomes of laparoscopic vs. open hepatectomies (LH and OH, respectively) with the surgical indication of HCC at left lateral liver sector were further compared and analyzed.

**Results:**

18 of 29 patients in the laparoscopic group and 75 patients in the conventional open group had primary HCC. The demographic data was essentially the same for the two groups. Statistical analysis revealed that the LH group had smaller tumor size, higher blood transfusion requirement, longer duration of inflow control and parenchymal transection, and longer operation time. However, no significant difference was observed in terms of complication rate, mortality rate, and hospital stay between the two groups. After adjusting for tumor size, LH and OH showed no statistical difference in the amount of blood transfusion, operation time and patient survival.

**Conclusions:**

This study demonstrated that stapleless LLS is a safe and feasible procedure for less experienced liver surgeons to resect HCC located at the left lateral liver sector. This stepwise stapleless LSS can not only achieve surgical results comparable to OH but also can provide a platform for liver surgeons to apply laparoscopic technique before conducting more complicated liver resections.

## Background

Laparoscopic surgery has been proven to be an effective surgical approach in many abdominal diseases, including acute cholecystitis, colon cancer, and gastroesophageal reflux disesase [1–3]. With improvements in surgical techniques and laparoscopic instruments, laparoscopic surgery has also shown promising results in major abdominal operations in recent decades. Laparoscopic liver resection, for example, has been shown to be a feasible and safe technique for hepatic tumors with surgical results comparable to conventional open hepatectomy [4, 5]. However, because the liver is a highly vascular solid organ, the resection of liver tumors still carries substantial risk of morbidity, especially in patients with liver cirrhosis [6]. In an attempt to guide liver surgeons worldwide, the first International Consensus Conference on Laparoscopic Liver Resections was thus convened and a statement formulated. In this so-called “Louisville Statement”, laparoscopic liver resection was considered a standard practice for liver tumors located in the left lateral liver sector [7]. Hence, laparoscopic left lateral sectionectomy (LLS) was to be performed by surgeons who had developed sufficient laparoscopic techniques. However, due to lack of strong evidence in case of hepatocellular carcinoma (HCC), this recommendation was not strongly supported by the second International Consensus Conference on Laparoscopic Liver Resections [8]. The fact that HCC may arise in the context of liver cirrhosis while most of the series published so far were based on evidence from colorectal liver metastasis or benign liver lesions leaves the status of laparoscopic liver resection for HCC undetermined.

A recent study published by a Hong Kong group demonstrated their long-term outcome for HCC. In their study, laparoscopic left lateral sectionectomy resulted in survival outcome comparable to the conventional open approach [9]. Their promising result was exhilarating. However, the operating surgeons in that study were all well-known and experienced liver surgeons. Their excellent results were not unexpected. The surgical outcome of less experienced surgeons or surgeons with lower case volumes, on the other hand, remains unknown. To further address this issue, we conducted the current study and aim to reappraise the Louisville statement by examining the outcome of LLS performed by a young liver surgeon.

## Methods

### Patients

From 2009 to 2017, records of patients who underwent standard left lateral sectionectomy at Chang Gung Memorial Hospital, Linkou, Taiwan, were retrospectively reviewed. Only patients who had histologically proven primary HCC were included in the final comparative study. The conventional open hepatectomies were performed by experienced liver surgeons in the same surgical department. Laparoscopic left lateral sectionectomy, on the other hand, was conducted by a young surgeon who had a special interest in the laparoscopic procedures. The index young surgeon had received a 5-year postgraduate training as a surgical resident at Linkou Chang Gung Memorial Hospital. The surgeon received, in addition to trainings in conventional open hepatobiliary surgeries, comprehensive training in fundamental laparoscopic procedures including laparoscopic cholecystectomy (LC) and laparoscopic appendectomy (LA) during his last two years of residency. Since the index surgeon had become a board-certified gastrointestinal surgeon in 2012, the LLS also started from 2012. Moreover, because the instruments required by laparoscopic liver resections were not reimbursed by health insurance in Taiwan, only patients who were able to afford the cost were assigned to the LLS group.

With the approval of the Institutional Review Boards of Chang Gung Memorial Hospital (CGMH IRB No: 201701574B0 and No: 201600359B0), the recruited patients’ clinicopathological data were retrieved from the prospectively collected database. Patients who did not have detailed preoperative/intraoperative clinical records, or who did not have regular postoperative out-patient follow-up were excluded from our study. The tumor staging of the current study was based on the AJCC TNM staging system for HCC [10].

Preoperative diagnosis of HCC was established by characteristic features on imaging by either triphasic computed tomography (CT), magnetic resonance imaging (MRI), hepatic arteriography, and/or a serum α-fetoprotein (AFP) level greater than 200 ng/ml. Resection criteria were constant over the entire study period, including a lack of cancerous thrombi in the main trunk of the portal vein, no distant metastasis to other organs, a technically operable main tumor in the preoperative evaluation, and a adequate liver functional reserve. Liver function was routinely assessed preoperatively by Child–Pugh classification and indocyanine green retention test. A previous study identified an indocyanine green retention at 15 min (ICG-15) of less than 14% as the safety limit for major hepatic resection [11]. In our institute, an ICG-15 ≤ 10% was the prerequisite for major hepatic resection.

For LLS, the patients were placed in the reversed Trendelenburg position. Most procedures were performed by two surgeons, with the index operating surgeon standing on the right side of the patient and assistant surgeon on the left. The video laparoscope was introduced via a 12 mm vertical incision at the supraumbilical region. Another 12 mm working port was created at the right subcostal area in line with the falciform ligament. Two more 5 mm assistant ports were introduced at the lateral aspect of the right subcostal area, and at about the left mid-clavicular line in the left subcostal area, respectively. The peritoneal cavity was inspected to confirm the absence of metastatic disease. Laparoscopic ultrasound was introduced via the 12 mm working port to locate the primary tumor, search for possible additional tumors at bilateral lobes, identify the location and patency of major vascular structures, and define the transection line. After ultrasonic evaluation, the round ligament and falciform ligament were divided by energy device. The energy devices used for tissue dissection or liver parenchymal transection were usually Harmonic scalpels (EthiconTM) or Thunderbeat dissectors (OlympusTM). During liver parenchymal transection, the central venous pressure was maintained as low as possible (around 5 mmHg) and pneumopeirtoneum was kept at 15 mmHg to reduce venous bleeding from the transected surface. The liver parenchyma was transected along the lateral border of the falciform ligament, and the portal pedicles supplying segment 3 and 2, small hepatic veins, and left hepatic vein were identified and ligated individually by double HEM-O-LOK (Teleflex). In the current study, no vascular staplers were employed for liver parenchymal transection. Upon completion of liver transection, the transected surface was meticulously examined for bleeding or bile leakge. Electrocauterization, hemoclips, or suture were applied whenever necessary. The resected specimen was delivered through a transverse incision created at the suprapubic area. A Jackson-Pratt drain was routinely placed at the left subphrenic space for postoperative drainage.

For conventional open left lateral sectionectomy, an upper midline and right subcostal incision was usually made. Intraoperative exploration by both manual palpation and ultrasonography was performed to define the extent of the tumor(s), the texture of liver parenchyma, any invasion of the portal or hepatic veins, and the size of future liver remnant. A low central venous pressure was maintained to reduce venous bleeding as for the laparoscopic approach. For either LLS or conventional open surgery, inflow control with Pringle’s maneuver was predetermined and applied according to individual surgeon’s discretion. Parenchymal transection was performed using either crush clamp technique or Cavitron ultrasonic surgical aspirator (CUSA) based on surgeon’s preference. Hemostasis was achieved and bile leakage meticulously repaired in each operation.

Patients were cared for and monitored postoperatively according to a protocolized approach published previously [6]. All patients received blood exams and triphasic CT study one to two months after the operation. Out-patient follow-up with serial lab tests and image study was arranged every 2–3 months after hospital discharge.

### Definition

Operation duration was defined as the time interval elapsed from anesthesia induction to extubation. Major surgical complications comprised grade III and IV surgical complications as described previously [6, 12]. Thirty-day mortality was defined as the occurrence of death within 30 days after the operation, and in-hospital mortality was defined as death during the same hospital stay. Recurrence was defined as the appearance of characteristic image findings during regular postoperative radiologic examinations. Early recurrence was defined as recurrence within two years of the initial curative operation [13]. Disease-free survival (DFS) was calculated from the date of surgery to the date of the first documented clinical disease recurrence. Overall survival (OS) was defined as the time elapsed from the date of surgery to either the date of death or the date of the last contact. Cases with surgical mortality, defined as death within one month of surgery, were excluded from the survival analyses.

### Statistical analysis

The statistical analysis was performed with IBM SPSS Statistics 21 (IBM Corporation, Software Group, Somers, NY, USA). Fisher’s exact test or Pearson’s χ2 test was used to analyze categorical data. Student’s t test was used to analyze continuous variables. Kaplan-Meier analysis and log-rank test were used to determine and compare the OS and DFS. Statistical significance was defined as *P* values < 0.05 in two-sided tests.

## Results

From 2012 to 2017, a total of twenty-nine LLS were performed by the index surgeon. The demographic data of these patients receiving LLS is summarized in Table 1. Almost half of the patients were older than 60 years old. The most common etiology was HCC (18 patients, LH group), followed by hepatic hemangioma, hepatic cysts, focal nodular hyperplasia (FNH), cholelithiasis, and amebic liver abscess. The size of the tumors was mostly less than 5 cm in diameter. Twenty-five patients (86.2%) received purely laparoscopic surgery, 3 had (10.3%) robotic surgery, and 1 (3.4%) underwent hybrid operation. No conversion laparotomy was encountered. Inflow control was employed in only 5 patients (17.2%). Surgical complication rate was 13.8%. For comparison, a further 75 patients who underwent conventional open left lateral sectionectomy for their primary HCC (OH group) from 2009 to 2017 were included for subsequent analysis.

**Table 1:** Demographic data of patients receiving laparoscopic left lateral sectionectomy by a single surgeon (*n* = 29)

Variables	(%)	Variables	(%)
Age ≦60 years	15 (51.7)	OP method	
Male gender	17 (58.6)	Pure laparoscopic	25 (86.2)
Disease entity		Hybrid	1 (3.4)
Hepatocellular carcinoma	18 (62.1)	Pure robotic	3 (10.3)
Hemangioma	5 (17.2)	Conversion laparotomy	0 (0)
Liver cysts	3 (10.3)	Inflow control	
Focal nodular hyperplasia	1 (3.4)	Pringle’s maneuver	5 (17.2)
Cholelithiasis	1 (3.4)	No inflow control	24 (82.8)
Amebic liver abscess	1 (3.4)	Duration of operation (hour) ^a^ (range)	4.63 ± 1.81 (2.3–10.4)
Tumor size (cm) (range 1.0–12.6 cm)		Duration of parenchymal transection ^a^ (minute) (range)	90.81 ± 52.78 (35–300)
≤ 2	5 (17.2)	Blood loss ^a^ (ml) (range)	179.53 ± 161.9 (20–600)
2–5	17 (58.6)	Complication (Yes)	4 (13.8)
> 5	7 (24.1)	Grade II/III/V	4 / 0 / 0

^a^ Mean ± standard deviation

As for HCC per se, the LH group and OH group shared similar clinical characteristics (Table 2). The rate of comorbid illness was comparable between the two groups. Hepatitis B virus (HBV) infection accounted for about 50% of cases, while around 35% of patients had chronic hepatitis C virus (HCV) infection. The BMI was 25.7 kg/m^2^ for the LH group and 25.3 kg/m^2^ for the OH group. In both groups, the vast majority of patients were Child-Pugh A. However, one-fourth of patients in the OH group were symptomatic upon presentation, in contrast to 0% in the LH group (*p* = 0.019). The median follow-up time was 35.8 months for the LH group and 37.1 months for the OH group.

**Table 2:** Comparison of clinical characteristics between laparoscopic stapleless left lateral sectionectomy (LH) and open left lateral sectionectomy (OH) for hepatocellular carcinoma

Categorical variables	LH group ^a^ (*n* = 18)	OH group (*n* = 75)	*p* value
Age (> 65 years (%))	9 (50.0)	27 (36.0)	0.293
Gender (Male(%) / Female(%))	13(72.2) / 5(27.8)	56(74.7) / 19(25.3)	1.000
Diabetes Mellitus (Yes (%))	6 (33.3)	18 (24.0)	0.549
Hypertension (Yes (%))	7 (38.9)	23 (37.7)	1.000
ESRD^b^ (Yes (%))	0 (0)	3 (4.0)	1.000
Smoking (Yes (%))	4 (22.2)	13 (17.3)	0.735
Alcohol (Yes (%))	7 (38.9)	14 (18.7)	0.112
HBV surface antigen (Positive (%))	10 (55.6)	36 (48.0)	0.608
Hepatitis C virus (Positive (%))	7 (38.9)	27 (36.0)	1.000
Child-Pugh Classification (A(%) / B(%))	18(100) / 0(0)	72(97.3) / 2(2.7)	1.000
Symptoms (Yes (%))	0 (0)	19 (25.3)	0.019
ICG-15 (> 10% (%))	8 (44.4)	23 (31.9)	0.407
Preoperative α-fetoprotein (> 15 ng/mL (%))	8 (44.4)	33 (44.0)	1.000
Continuous variables ^c^	LH group ^a^ (n = 18)	OH group (n = 75)	*p* value
Age (years)	60.2 ± 3.24	61.4 ± 1.32	0.707
BMI (kg/m^2^)	25.7 ± 1.00	25.3 ± 0.42	0.704
ICG-15 (%)	10.4 ± 1.90	10.5 ± 1.25	0.970
Hemoglobin (g/dL)	13.3 ± 0.51	13.3 ± 0.25	0.998
Albumin (g/dL)	4.0 ± 0.11	4.1 ± 0.06	0.423
Bilirubin total (mg/dL)	0.54 ± 0.06	0.72 ± 0.05	0.122
Preoperative α-fetoprotein (ng/mL)	1496.4 ± 1356.6	1147.4 ± 42.5	0.748

^a^include laparoscopic and robotic left lateral sectionectomy

^b^end-stage renal disease

^c^mean ± standard error of mean

As for surgical variables, the LH group had significantly lower rate of inflow control when compared to the OH group (27.8% vs. 63.8%, *p* = 0.008). However, higher blood transfusion requirement, longer duration of inflow control and parenchymal transection, and longer operation time were observed in the LH group. No significant difference was found in terms of complication rate, mortality rate, and hospital stay. The early recurrence rate was also comparable between the two groups (Table 3).

**Table 3:** Comparison of surgical variables and outcome between laparoscopic stapleless left lateral sectionectomy (LH) and open left lateral sectionectomy (OH) for hepatocellular carcinoma

Categorical variables	LH group ^a^ (n = 18)	OH group (n = 75)	*p* value
Inflow control (Yes (%))	5 (27.8)	44 (63.8)	0.008
Blood transfusion (Yes(%))	3 (16.7)	1 (1.4)	0.023
Complications (Yes (%))	6 (33.3)	22 (29.3)	0.778
Major complications ^b^ (Yes (%))	0 (0)	9 (12)	0.198
Thirty-day mortality (Yes (%))	0 (0)	0 (0)	N.A.
In-hospital mortality (Yes (%))	0 (0)	1 (1.3)	1.000
Early recurrence ^c^(Yes (%))	3 (16.7)	30 (40.0)	0.098
Continuous variables ^d^	LH group ^a^ (n = 18)	OH group (n = 75)	*p* value
Operative duration (minutes)	287.0 ± 28.91	221.4 ± 8.00	0.041
Blood loss (ml)	217.2 ± 45.00	239.8 ± 40.58	0.794
Duration of inflow control ^e^ (minutes)	85.6 ± 8.04	35.0 ± 3.16	< 0.001
Duration of parenchymal transection (minutes)	96.7 ± 15.98	53.8 ± 3.80	0.018
Post-OP hospital stay (days) (range)	8.44 ± 0.54 (5–15)	9.7 ± 0.50 (6–29)	0.238

^a^include laparoscopic and robotic left lateral sectionectomy

^b^major surgical complications include grade III-IV surgical complications

^c^recurrence within two years after the index operation

^d^mean ± standard error of mean

^e^mean duration among those who had inflow control

The pathological characteristics are summarized in Table 4. The tumor size in the LH group was significantly smaller than that in the OH group (mean tumor size: 3.2 cm vs. 4.9 cm, *p* = 0.011). The OH group had a slightly higher rate of tumor rupture when compared to the LH group (17.3% vs. 0%, *p* = 0.066). Negative resection margin was achieved in every patient in the LH group and in all but one patient in the OH group. In addition to 100% R0 resection rate, more than 60% of the LH group had their safety margin larger than 1 cm in width. Histologically-proven liver cirrhosis was present in 44 and 58% of the LH and OH groups, respectively. The other pathological parameters were essentially the same between the two groups.

**Table 4:** Comparison of pathologic characteristics between laparoscopic stapleless left lateral sectionectomy (LH) and open left lateral sectionectomy (OH) for hepatocellular carcinoma

Variables	LH group ^a^ (n = 18)	OH group (n = 75)	*p* value
Tumor size (cm)^b^	3.2 ± 0.50	4.9 ± 0.41	0.011
Tumor size (> 5 cm (%))	2 (11.1)	26 (34.7)	0.083
Encapsulation (Yes (%))	16 (88.9)	64 (85.3)	1.000
Capsular invasion (Yes (%))	12 (66.7)	45 (60.8)	0.789
Tumor rupture (Yes (%))	0 (0)	13 (17.3)	0.066
Vascular invasion (Yes (%))	5 (27.8)	20 (26.7)	1.000
Daughter nodules (Yes (%))	2 (11.1)	9 (12.0)	1.000
Resection margin (Negative (%))	18 (100)	74 (98.7)	1.000
Safety margin (≥1 cm (%))	11 (61.1)	35 (46.7)	0.304
Edmonson and Steiner grade (III and IV (%))	6 (35.3)	27 (37.0)	1.000
Cirrhosis (Yes (%))	8 (44.4)	44 (58.7)	0.302
T stage			
T1 (%)	11 (61.1)	37 (52.9)	0.631
T2 (%)	4 (22.2)	15 (21.4)	
T3a/T3b (%)	0/4 (0/5.7)	1/2 (1.4/11.1)	
T4 (%)	1 (5.6)	13 (18.6)	

^a^include laparoscopic and robotic left lateral sectionectomy

^b^mean ± standard error of mean

**Fig. 1:**
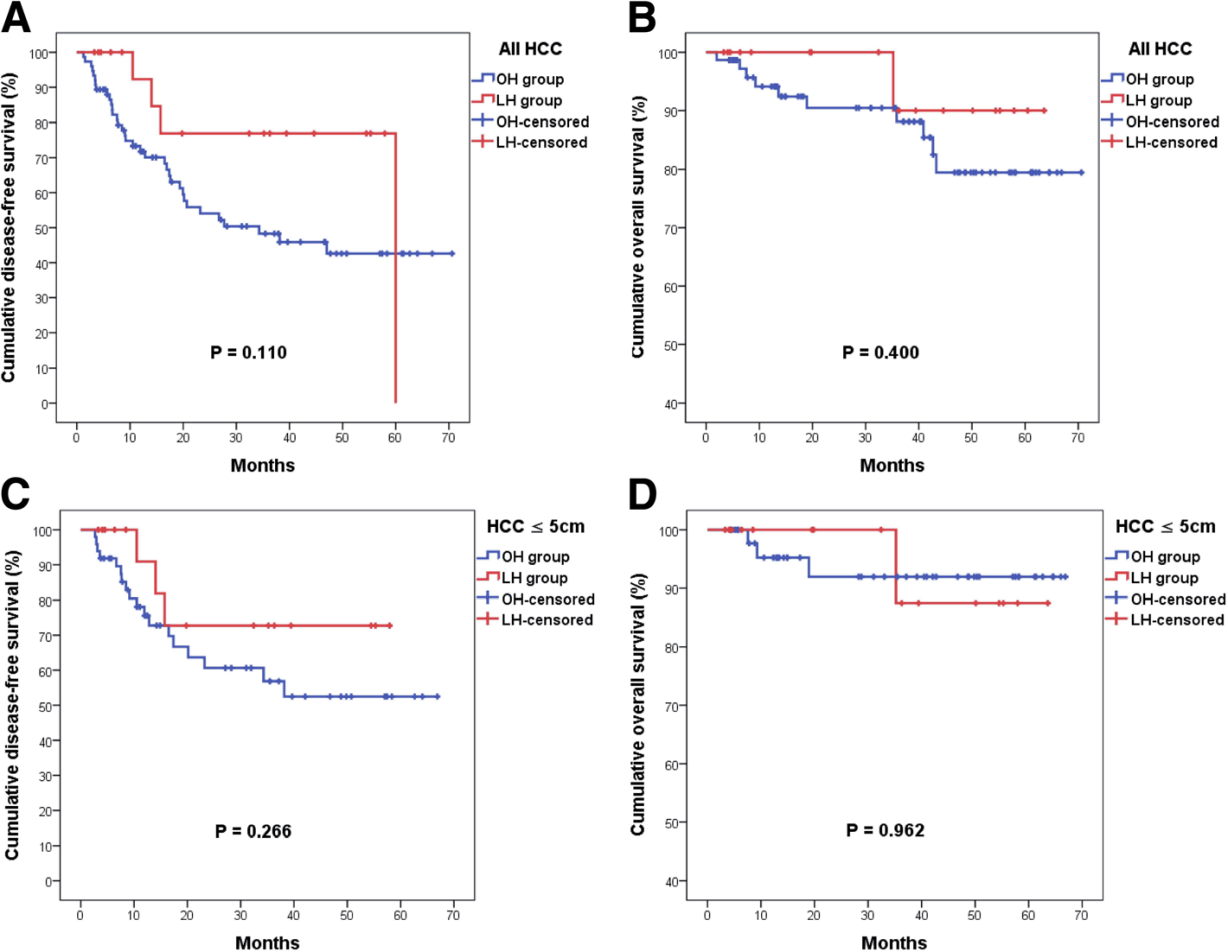
Kaplan–Meier disease-free survival (DFS) curves and overall survival (OS) curves for hepatocellular carcinoma treated by LH or OH. (**a** and **b**, all HCC) The mean DFS was 49.25 months for the LH group and 39.24 months for the OH group (*P* = 0.110). The mean OS was 60.73 months for the LH group and 61.58 months for the OH group (*P* = 0.400). Laparoscopic liver resection for HCC located at left lateral liver sector can achieve satisfactory oncological outcome when compared to the conventional open surgery. (**c** and **d**, HCC less than 5 cm in diameter). The mean DFS was 45.80 months for the LH group and 42.46 months for the OH group (*P* = 0.266). The mean OS was 60.02 months for the LH group and 62.51 months for the OH group (*P* = 0.962). Laparoscopic hepatectomy can achieve comparable oncological outcome when compared to the conventional open surgery, especially for smaller HCC

The oncological survival outcome has been illustrated in Fig. 1a-b. The mean disease-free survival (DFS) was 49.25 ± 6.29 months for the LH group and 39.24 ± 3.67 months for the OH group (*P* = 0.110). The mean overall survival (OS) was 60.73 ± 2.70 months for the LH group and 61.58 ± 2.61 months for the OH group (*P* = 0.400). Laparoscopic surgery can achieve satisfactory oncological outcome when compared to conventional open surgery.

For more matched analysis, patients with HCC less than 5 cm in diameter were included for subgroup analysis. The clinical backgrounds were comparable between the two groups and are summarized in Table 5. After adjusting for tumor size, the LH group still had lower rate of inflow control than the OH group. However, the rate of blood transfusion became similar when smaller tumors were concerned. In addition, despite longer duration of inflow control and parenchymal transection, the total operative duration was comparable between the two groups. There were still no significant differences found in terms of complication rate, mortality rate, hospital stay, or early recurrence rate (Table 6). The pathological variables in the adjusted cohort are summarized in Table 7. Like the original cohort, more than 60% of the LH group had their safety margin larger than 1 cm in width. The other pathological characteristics remain similar between the two groups. The oncological outcome has been illustrated in Fig. 1c-d. The mean DFS was 45.80 ± 5.99 months for the LH group and 42.46 ± 4.36 months for the OH group (*P* = 0.266). The mean OS was 60.02 ± 3.32 months for the LH group and 62.51 ± 2.43 months for the OH group (*P* = 0.962). Laparoscopic hepatectomy can achieve comparable oncological outcome when compared to conventional open surgery, especially for smaller HCC.

**Table 5:** Comparison of clinical characteristics between laparoscopic stapleless left lateral sectionectomy (LH) and open left lateral sectionectomy (OH) for hepatocellular carcinoma less than 5 cm

Categorical variables	LH group ^a^ (*n* = 16)	OH group (*n* = 49)	*p* value
Age (> 65 years (%))	8 (50.0)	15 (30.6)	0.229
Gender (Male(%) / Female(%))	12(75.0) / 4(25.0)	35(71.4) / 14(28.6)	1.000
Diabetes Mellitus (Yes (%))	5 (31.3)	15 (30.6)	1.000
Hypertension (Yes (%))	5 (31.3)	14 (33.3)	1.000
ESRD^b^ (Yes (%))	0 (0)	1 (2.0)	1.000
Smoking (Yes (%))	4 (25.0)	10 (20.4)	0.732
Alcohol (Yes (%))	7 (43.8)	13 (26.5)	0.223
HBV surface antigen (Positive (%))	8 (50.0)	24 (49.0)	1.000
Hepatitis C virus (Positive (%))	9 (56.3)	29 (59.2)	1.000
Child-Pugh Classification (A(%) / B(%))	16(100) / 0(0)	48(100) / 0(0)	N.A.
Symptoms (Yes (%))	0 (0)	9 (18.4)	0.098
ICG-15 (> 10% (%))	8 (50.0)	16 (34.8)	0.374
Preoperative α-fetoprotein (> 15 ng/mL (%))	7 (43.8)	24 (49.0)	0.779
Continuous variables ^c^	LH group ^a^ (*n* = 16)	OH group (*n* = 49)	*p* value
Age (years)	60.1 ± 3.61	61.1 ± 1.33	0.790
BMI (kg/m^2^)	25.8 ± 1.10	25.5 ± 0.55	0.837
ICG-15 (%)	11.0 ± 2.09	9.78 ± 1.04	0.575
Hemoglobin (g/dL)	13.6 ± 0.52	13.5 ± 0.30	0.963
Albumin (g/dL)	4.1 ± 0.12	4.2 ± 0.05	0.101
Bilirubin total (mg/dL)	0.56 ± 0.07	0.60 ± 0.04	0.655
Preoperative α-fetoprotein (ng/mL)	150.1 ± 76.9	1196.9 ± 588.6	0.316

^a^include laparoscopic and robotic left lateral sectionectomy

^b^end-stage renal disease

^c^mean ± standard error of mean

**Table 6:** Comparison of surgical variables and outcome between laparoscopic stapleless left lateral sectionectomy (LH) and open left lateral sectionectomy (OH) for hepatocellular carcinoma less than 5 cm

Categorical variables	LH group ^a^ (n = 16)	OH group (n = 49)	*p* value
Inflow control (Yes (%))	5 (31.3)	27 (57.4)	0.088
Blood transfusion (Yes(%))	2 (12.5)	1 (2.0)	0.147
Complications (Yes (%))	5 (31.3)	13 (26.5)	0.753
Major complications ^b^ (Yes (%))	0 (0)	2 (4.1)	1.000
Thirty-day mortality (Yes (%))	0 (0)	0 (0)	N.A.
In-hospital mortality (Yes (%))	0 (0)	0 (0)	N.A.
Early recurrence ^c^(Yes (%))	3 (18.8)	16 (32.7)	0.357
Continuous variables ^d^	LH group ^a^ (n = 16)	OH group (n = 49)	*p* value
Operative duration (minutes)	267.1 ± 23.96	219.1 ± 10.04	0.079
Blood loss (ml)	216.3 ± 50.33	172.5 ± 26.47	0.449
Duration of inflow control ^e^ (minutes)	85.6 ± 8.04	32.8 ± 3.51	< 0.001
Duration of parenchymal transection (minutes)	80.5 ± 9.30	50.5 ± 4.20	0.001
Post-OP hospital stay (days) (range)	8.4 ± 0.63 (5–15)	9.1 ± 0.54 (6–29)	0.474

^a^include laparoscopic and robotic left lateral sectionectomy

^b^major surgical complications include grade III-IV surgical complications

^c^recurrence within two years after the index operation

^d^mean ± standard error of mean

^e^mean duration among those who had inflow control

**Table 7:** Comparison of pathologic characteristics between laparoscopic stapleless left lateral sectionectomy (LH) and open left lateral sectionectomy (OH) for hepatocellular carcinoma less than 5 cm

Variables	LH group ^a^ (n = 16)	OH group (n = 49)	*p* value
Tumor size (cm)^b^	2.6 ± 0.27	2.9 ± 0.15	0.350
Encapsulation (Yes (%))	14 (87.5)	44 (89.8)	1.000
Capsular invasion (Yes (%))	10 (62.5)	30 (62.5)	1.000
Tumor rupture (Yes (%))	0 (0)	2 (4.1)	1.000
Vascular invasion (Yes (%))	4 (25.0)	13 (26.5)	1.000
Daughter nodules (Yes (%))	2 (12.5)	6 (12.2)	1.000
Resection margin (Negative (%))	16 (100)	48 (98.0)	1.000
Safety margin (≥1 cm (%))	10 (62.5)	27 (55.1)	0.773
Edmonson and Steiner grade (III and IV (%))	4 (26.7)	17 (36.2)	0.551
Cirrhosis (Yes (%))	8 (50.0)	32 (65.3)	0.376
T stage			
T1 (%)	11 (68.8)	28 (59.6)	0.816
T2 (%)	4 (25.0)	14 (29.8)	
T3a/T3b (%)	0/1 (0/6.3)	0/3 (0/6.4)	
T4 (%)	0 (0)	2 (4.3)	

^a^include laparoscopic and robotic left lateral sectionectomy

^b^mean ± standard error of mean

The influence of liver cirrhosis on laparoscopic hepatectomy was investigated and is summarized in Table 8. There was no significant differences found in terms of rate of inflow control, blood transfusion, complications, mortality, or early recurrence between cirrhotic and non-cirrhotic groups. The duration of parenchymal transection and total operative duration were also comparable between the two groups. However, the cirrhotic group tended to have more operative blood loss and longer postoperative hospital stay than the non-cirrhotic group.

**Table 8:** Comparison of surgical variables and outcome between cirrhotic and non-cirrhotic livers when performing laparoscopic stapleless left lateral sectionectomy for hepatocellular carcinoma

Categorical variables	Cirrhotic (*n* = 8)	Non-cirrhotic (*n* = 10)	*p* value
Inflow control (Yes (%))	3 (37.5)	2 (20.0)	0.608
Blood transfusion (Yes(%))	2 (25.0)	1 (10.0)	0.559
Complications (Yes (%))	4 (50.0)	2 (20.0)	0.321
Major complications ^a^ (Yes (%))	0 (0)	0 (0)	N.A.
Thirty-day mortality (Yes (%))	0 (0)	0 (0)	N.A.
In-hospital mortality (Yes (%))	0 (0)	0 (0)	N.A.
Early recurrence ^b^(Yes (%))	1 (12.5)	2 (20.0)	1.000
Continuous variables ^c^	Cirrhotic (*n* = 8)	Non-cirrhotic (*n* = 10)	*p* value
Operative duration (minutes)	270.9 ± 24.47	299.9 ± 49.28	0.607
Blood loss (ml)	321.3 ± 80.99	134.0 ± 32.70	0.060
Duration of inflow control^d^ (minutes)	75.7 ± 9.24	100.5 ± 4.50	0.139
Duration of parenchymal transection (minutes)	82.8 ± 9.93	105.0 ± 25.06	0.521
Post-OP hospital stay (days) (range)	9.6 ± 0.96 (6–15)	7.5 ± 0.52 (5–10)	0.057

^a^major surgical complications include grade III-IV surgical complications

^b^recurrence within two years after the index operation

^c^mean ± standard error of mean

^d^mean duration among those who had inflow control

## Discussion

HCC is the most common primary malignancy of the liver and causes more than 8000 deaths each year in Taiwan [14, 15]. With improvements in patient selection, surgical instruments, operative techniques, and postoperative care, the mortality rate of curative surgical resection has improved dramatically in recent decades [16–19]. According to a recent study, the 30-day mortality rate was only 1.8% and the in-hospital mortality rate was 2.9% after hepatectomy for HCC [6, 20]. Minimally invasive liver resection was thus developed in the late 1990s after significant improvements in surgical outcome [21]. It has received worldwide acknowledgement and more and more liver surgeons have started to perform laparoscopic liver resections during the last decade. According to a review article in 2009, as many as 2804 minimally invasive liver resections were conducted for either benign or malignant liver diseases in the early twenty-first century [5]. Due to this widespread acceptance, the First World Consensus Conference on Laparoscopic Liver Surgery suggested laparoscopic liver resection become standard practice for lesions located at the left lateral liver sector [7]. This statement has encouraged liver surgeons to devote themselves to conducting laparoscopic left lateral sectionectomy. Nevertheless, this recommendation failed to gain full support from the juries at the Second International Consensus Conference on Laparoscopic Liver Resection. The fact that most of the evidence presented for LLS were from series of colorectal liver metastasis rendered this recommendation less convincing [8]. For this reason, many studies have since been conducted to investigate the result of LLS for HCC. In these series, LLS has been shown to have surgical morbidity and mortality rates comparable to the open approach [9, 22–24]. Nevertheless, most studies failed to compare the long-term oncological outcome between laparoscopic approach and conventional open approach. In addition, since most studies were obtained from operations performed by experienced and authorative liver surgeons, their results may not be fully applicable to the “real world” scenario. Our study, in which all of the LLS was performed by a single young surgeon, may be the first one in the English literature to provide strong evidence for beginners or hospitals with lower case volumes to perform this operation for HCC.

In the current study, we demonstrated that for HCC located in the left lateral liver sector, laparoscopic liver resection provided results comparable to the conventional open approach in terms of blood loss, surgical complication rate, mortality rate, and early recurrence rate. The total operative duration was also similar between the two approaches when smaller tumors were concerned. Moreover, the presence of liver cirrhosis did not affect the results of laparoscopic liver resection, in that the amount of blood products transfused, surgical complication rate, mortality, and early recurrence rate were not different between the cirrhotic and non-cirrhotic groups. In addition to excellent surgical result, the surgical radicality was not compromised by the laparoscopic approach, whereby all patients in the laparoscopic group had an R0 resection and more than 60% of patients had their safety margin larger than 1 cm in width. This result was encouraging since the most prevailing doubt regarding laparoscopic cancer surgery is tumor radicality! The current study demonstrated that laparoscopic left lateral sectionectomy can provide complete HCC eradication, just as in the conventional open approach, even in the cirrhotic liver. Furthermore, regardless of the tumor size, the long-term oncological survival after LLS was equivalent to that after open surgery. Our study, as a result, is one of the first report in the English literature to demonstrate the surgical as well as oncological outcome of LLS for HCC. Given the inherent merits of smaller wounds, less pain, better cosmetics, and earlier postoperative ambulation and recovery, laparoscopic left lateral sectionectomy should be the standard treatment for HCC even when performed by less experienced surgeons. The recommendation concluded by the Louisville statement thus stands reappraised and validated [7].

The technique employed in the current study did not encourage the use of vascular staplers for parenchymal transection during LLS. Since the left lateral liver sector is usually thin and contributes only about 15–30% of total liver volume, the resection of this sector rarely results in postoperative hepatic failure. The relatively constant vascular anatomy and straight transection plane also render this operation less challenging to hepatobiliary surgeons [9]. Many liver surgeons, whether experienced or beginners, would thus apply vascular staplers for parenchymal transection during LLS in order to facilitate the operation. However, we hold the view that since the anatomy of the left lateral sector is constant and straight, it is a good opportunity for liver surgeons to familiarize themselves with the techniques required to perform laparoscopic liver resection. We believe this stapleless approach is a safe and efficient opportunity for liver surgeons to gather the experience necessary to overcome the learning curve required for LH. This concept is similar to that established by Komatsu et al. [25]. Last but not the least, we found that laparoscopic Cavitron ultrasonic surgical aspirator (CUSA) was rarely indicated in LLS since the other energy devices such as Harmonic scalpels or Thunderbeat dissectors are capable enough to complete the parenchymal transection. This result is also comparable to that published by Liu et al. in 2017 [26]. Although our stapleless technique appears promising, some drawbacks still require considerations. First, to skeletonize the portal pedicles and hepatic veins, more time is necessary for such meticulous parenchymal transection in order to complete the entire operation. Moreover, to achieve such extensive dissection, the energy device would produce some smoke, which may blur the video laparoscope and hamper the operation. However, we believe these are only minor flaws and would not alter our commitment towards the stapleless LLS. As a result, through this stapleless stepwise approach, we provide liver surgeons with an opportunity to practice their techniques in preparation for more complicated major liver resections.

In the current study, the postoperative hospital stay was not significantly different between the two groups. We believe this may be attributed to several reasons. First, since we just started our laparoscopic program, our immature technique may result in prolonged postoperative stay. The lack of knowledge regarding post-laparoscopic recovery and care may also have resulted in delayed hospital discharge. Second, since our national health care insurance reimburses the cost of postoperative hospital stay, patients usually prefer not to be discharged until they have completely recovered. Lastly, the small number of patients in the current study renders the statistics less significant. We believe the trend towards shorter hospital stay for LLS will become more pronounced when more patients have accumulated.

The current study compared the outcome after laparoscopic and conventional open left lateral sectionectomy for HCC. It is often difficult to initiate a new surgical technique, especially when there has been an long-established equivalent counterpart. For minimally invasive surgery (MIS) per se, during the residency years of the index young surgeon, MIS in Taiwan were mostly limited to simple procedures such as LC, LA, and laparoscopic gastrorrhaphy. Laparoscopic major gastrointestinal or hepatobiliary surgeries, on the other hand, were relatively rare and performed mainly by several experienced surgeons. Thanks for the support from the institutions and related surgical associations in Taiwan, the index surgeon and other motivated surgeons, after finishing their residency training, gained access to more complicated laparoscopic procedures including laparoscopic hepatectomy. Almost a decade after, MIS in Taiwan is a booming technique that almost every medical center now is capable of performing laparoscopic gastrointestinal surgeries. Residents nowadays are able to observe and participate in more complicated laparoscopic procedures during their training period. Given the evidence obtained from previous study and the current research [27], senior residents or less experienced surgeons will have the opportunity to perform LLS as the first step toward laparoscopic hepatectomy.

Despite encouraging results, the current study still has some limitations. First, since it is a retrospective study based on clinical data retrieved from a database, incomplete data collection is inevitable when reviewing records many years ago. Second, the lack of randomization between LH and OH groups also introduced selection bias into our final statistical analysis. A prospective randomized control trial is thus warranted to validate our findings. Third, more than one surgeon conducted the conventional open hepatectomy, the results after OH may be less homogenous. Fourth, in some cases, the follow-up duration was not long enough. A longer follow-up period is thus required to give a more convincing result. Lastly, as mentioned above, we need more laparoscopic experience to demonstrate the significance of LH for HCC.

## Conclusions

The current study demonstrated that our stapleless laparoscopic liver resection is a safe and feasible procedure for less experienced liver surgeons to resect HCC located at the left lateral liver sector, even for HCC in cirrhotic livers. It delivers comparable surgical results with similar operation time and blood loss, less need for inflow control, low complication rate, and zero mortality rate. The oncological disease-free survival and overall survival rates are also equivalent to the conventional open approach. As suggested by the Louisville statement, LLS should be the standard treatment of choice for HCC, especially when the tumor is less than 5 cm in diameter. In addition, our stapleless LLS can provide a platform for liver surgeons to apply laparoscopic technique before conducting more complicated liver resections. Further randomized prospective studies are warranted to determine the actual role of laparoscopic surgery in the treatment of HCC.
